# A Contemporary Multifaceted Insight into the Association Between Diabetes Mellitus and Diverticular Disease: An Update About Geriatric Syndrome

**DOI:** 10.3390/geriatrics10010030

**Published:** 2025-02-18

**Authors:** Ridwan Hashi, Rahma Thamer, Ahmed Hassan, Khalid Canna, Musaab Ahmed, Mohamed T. Hassan, Safaa Badi, Mohamed H. Ahmed

**Affiliations:** 1 Medical School, The University of Buckingham, Buckingham MK18 1EG, UK; 2007239@buckingham.ac.uk; 2 Department of Trauma and Orthopaedic, Oxford University Hospital, Oxford OX3 9DU, UK; rahma.thamer@ouh.nhs.uk; 3 Faculty of Medicine, Alexandria University, Alexandria 5424041, Egypt; ahmed.mohamed2133@alexmed.edu.eg; 4 Department of Surgery, Bedford Hospital NHS Trust, Bedfordshire MK42 9DJ, UK; khalid.canna@bedfordhospital.nhs.uk; 5 College of Medicine, Ajman University, Ajman P.O. Box 346, United Arab Emirates; m.omer@ajman.ac.ae; 6 Center of Medical and Bio-Allied Health Sciences Research, Ajman University, Ajman P.O. Box 346, United Arab Emirates; 7 Kasr Alainy Faculty of Medicine, Cairo University, Cairo 12613, Egypt; mohamed_tar_hassan@students.kasralainy.edu.eg; 8 Department of Clinical Pharmacy, Faculty of Pharmacy, Omdurman Islamic University, Khartoum 11111, Sudan; safaabadi30@gmail.com; 9 Department of Geriatric Medicine, Milton Keynes University Hospital NHS Foundation Trust, Eaglestone, Milton Keynes MK6 5LD, UK; 10 Department of Medicine and HIV Metabolic Clinic, Milton Keynes University Hospital NHS Foundation Trust, Eaglestone, Milton Keynes MK6 5LD, UK; 11 Honorary Senior Lecturer of the Faculty of Medicine and Health Sciences, University of Buckingham, Buckingham MK18 1EG, UK

**Keywords:** diabetes mellitus, diverticular disease, obesity, insulin resistance

## Abstract

**Introduction:** Diverticular disease, once considered a rare geriatric gastrointestinal condition, has now become a prevalent disorder associated with increased morbidity and healthcare costs. The spectrum of complications from diverticular disease ranges from incidental findings to more serious issues such as bleeding and diverticulitis. Symptomatic diverticular disease represents a significant economic burden in the western world. Diabetes mellitus is a major global health issue. As global aging accelerates, geriatric syndromes such as diabetes mellitus (DM) and diverticular disease (DD) are becoming increasingly prevalent. Understanding their interplay is critical, particularly within the geriatric population. Both conditions are linked to lifestyle, dietary habits, and changes in gut physiology. Additionally, age-related alterations in the gut microbiome and immune system make this association more complex, contributing to morbidity and healthcare burdens in older adults. The primary aim of this review is to provide an update on the association between diabetes mellitus and diverticular disease. **Methods:** This narrative review explores the association between diabetes mellitus and diverticular disease. Relevant articles were identified by searching major databases. **Results:** Risk factors for diverticular disease include insulin resistance, diabetes mellitus, smoking, non-alcoholic fatty liver disease, lack of physical activity, a low-fibre diet, and a high-carbohydrate diet. These risk factors are also associated with the development of diabetes mellitus. Major population studies indicate that diabetes can either increase the risk of diverticular disease or have a neutral impact. A complication of diabetes mellitus includes impaired intestinal peristalsis and enteric nervous system dysfunction, which can ultimately lead to the formation of intestinal diverticula. High-calorie foods low in fibre are a staple in the diets of many type 2 diabetes mellitus patients, contributing to gut dysbiosis. A detrimental consequence of dysbiosis is a breach in the protective intestinal barrier, which promotes the development of diverticulosis. **Conclusions:** Diabetes mellitus may be associated with diverticular disease, and the risk factors that contribute to diabetes mellitus can also be linked to diverticular disease. Further studies are needed to explore the complex relationship between diabetes mellitus and diverticular disease.

## 1. Introduction

Diverticular disease, once a rare geriatric gastrointestinal condition, has now become a prevalent disorder that is associated with increased morbidity and healthcare costs [1]. The spectrum of complications associated with diverticular disease ranges from incidental findings to complicated bleeding and diverticulitis. Symptomatic diverticular disease is a major economic burden in the western world, with over 2 million cases in the United States in 1998; and the number is still increasing [2]. The UK follows a similar pattern, with up to 160,775 hospitalizations for diverticulitis occurring in the year 2019–2020 [3]. The precise prevalence of diverticular disease is difficult to confirm, as 80–85% of affected patients remain asymptomatic. However, recent estimates using colonoscopy and computed tomography (CT) data indicate that fewer than 5% of the 80–85% continue to develop diverticulitis and 12% continue to present with complicated diverticulitis [1,4]. The prevalence of diverticulosis increases with age and supports the hypothesis of diverticular disease being once considered a condition of the elderly. Diverticulosis affects 85% of the population over the age of 80, in comparison to 5–10% of the population over the age of 40 [5]. Whilst the earlier literature focused on incidence in the elderly population, recent studies suggest that the incidence rate of diverticulosis and thus diverticular disease has recently increased in younger age groups, particularly the 18–44 age group, where the incidence of diverticulosis increased from 0.151 to 0.251 per 1000 in only 7 years [6]. A population study performed in the United States used nationwide inpatient samples from 1998 to 2005 and analysed 267,000 patients admitted for acute diverticulitis. This study observed increased rates of admission in the 18–44 age group in comparison with older patients (82% vs. 36% respectively) [7]. Multiple studies have also considered whether a gender variation is also observed between different age groups post developing diverticulitis; these studies concluded that women are primarily more affected in the above-50 age group whereas men are more affected in the under-50 age group [8,9].

Aside from the variation in prevalence observed between age groups and gender, there is also a stark difference observed in prevalence between western and eastern nations. The highest prevalence is observed in the USA, Australia and Europe, where approximately 50% of the population over the age of 60 are diagnosed with diverticulosis [10]. This is in contrast to the incidence rates observed in Africa and Asia, where prevalence rates are observed to be less than 0.5% [10]. It remains unclear whether the low prevalence observed in the developing countries is due to limited access to healthcare—and thus health monitoring—or due to differences in lifestyles and comorbidity patterns. The latter is supported by the increased prevalence of diverticular diseases observed in the same populations after emigration to western countries [11]. In addition to the geographical variability observed between prevalence rates, there is also variance in the anatomical location of the diverticula in people affected with diverticular disease. It has been well demonstrated that in western countries the location of the diverticula is within the left sided and primarily within the sigmoid colon, whereas within Asian populations it is primarily within the right side of the colon [8]. In this mini-review article, we will review the evidence for the association between diabetes mellitus and diverticular disease.

## 2. Methods

**Figure 1:**
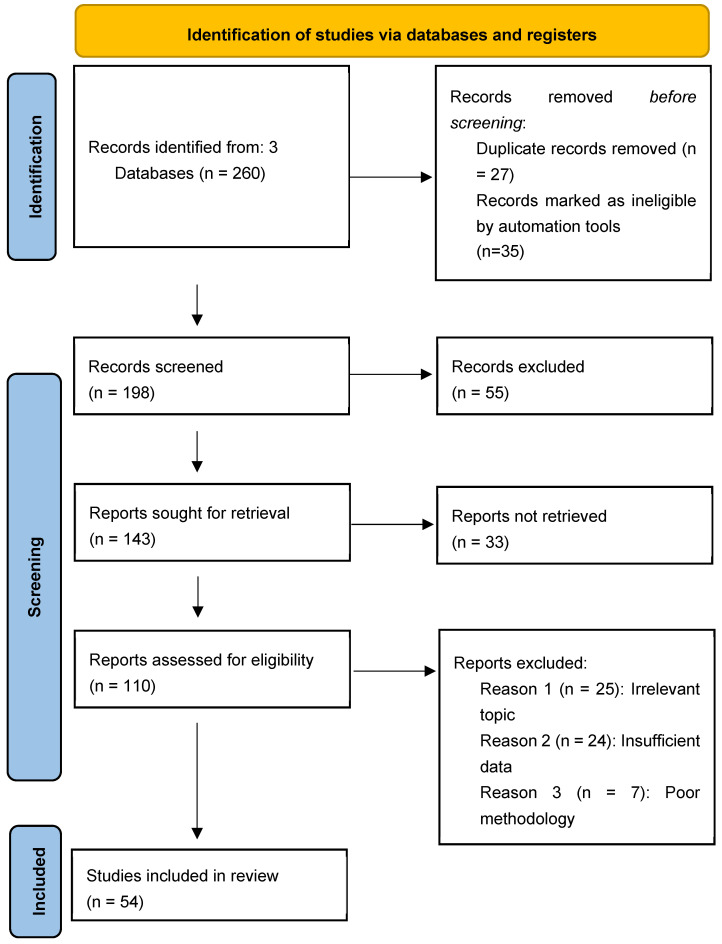
The methods used in searching the research databases.

In this narrative mini-review, a review of the literature was conducted and the following electronic databases were searched: Scopus, PubMed, Medline, Cochrane, and Google Scholar. The literature search process used a combination of keywords to ensure comprehensive coverage of relevant studies. The keywords used in the database search were as follows: (“diabetes mellitus” OR “diverticular disease”) (“diabetes mellitus” OR “type 1 diabetes” OR “type 2 diabetes mellitus “OR “hyperglycaemia”) (“diverticular disease” OR “diverticulosis”). After identifying the locations of the required articles, some of them were retrieved electronically and some were retrieved manually, by searching the libraries in our hospitals. Studies were included if they were published in English. We have included 45 articles, as this area not well researched (Figure 1).

### 2.1. Risk Factors

**Figure 2:**
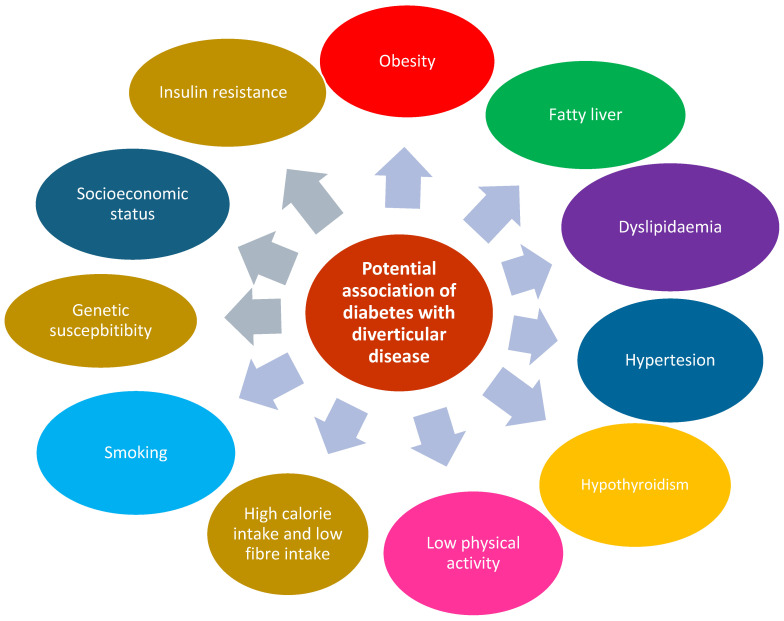
A representation of the most prevalent risk factors linked to diverticular disease. A more comprehensive explanation and detailed breakdown of these findings can be found in Table 1.

Several risk factors are thought to be linked with diabetes mellitus and diverticular disease. Epidemiological studies have explored the relationship between modifiable risk factors and developing diverticular disease. Among these factors are low fibre intake, red-meat-rich diet, high BMI and the existence of other co-morbidities such as diabetes mellitus and hypertension. Many of the previous studies have provided robust evidence that smoking and high BMI index increased the risk of diverticular disease; however, few worked to establish an association between dietary, environmental and behavioural factors in developing diverticulosis [1]. Of those is a study that compared multiple lifestyle factors to assess the risk of developing diverticulitis, which concluded that low-risk lifestyles decrease the risk of diverticular disease by 75%. They defined a low-risk lifestyle as one in which the consumption of red meat was fewer than four times a week, two hours of vigorous activity per week, BMI index of 18–24.9, consumption of at least 23 g of fibre per day and no history of smoking [12] (Figure 2 and Table 1).

A meta-analysis using six cohort studies assessed the association between obesity and, in particular, central obesity and suggested a positive linear relationship in men, with 28% increased risk of diverticular disease per 5 kg/m^2^ [25]. This is further strength-ened by the reduced risk of diverticulitis associated with high-fibre-intake diets that are high in fruits and vegetables [1]. Modern studies looking into modifiable risk factors are in concordance with the findings of Kopylov et al., who suggested that patients of higher age, high BMI and hypothyroidism were more likely to develop diverticulosis and thus diver-ticular disease [24]. The correlation in western nations having increased prevalence of di-verticular disease and being traditionally linked to low fibre intake, smoking and obesity has dominated most of the research, which is why vital research into the role of other metabolic syndromes on diverticular is scarce.

Non-alcoholic fatty liver disease, which is currently known as metabolic dysfunc-tion-associated steatotic liver disease (MASLD), is associated with high risk of diabetes mellitus and insulin resistance [26]. Pantic et al. showed that the presence of type 2 diabe-tes mellitus, insulin resistance, hypotriglyceridaemia, inflammation and low high-density lipoprotein-cholesterol were prominent in patients diagnosed with colonic diverticulosis and concomitant MASLD. They found that T2DM, hypothyroidism and hypertension are the main risk factors. Their conclusion is that hepatic steatosis was more commonly de-tected in more severe forms of colonic diverticulosis [27]. Sahin et al. concluded that in Turkish patients, there were lower rates of hepatosteatosis among elderly patients with diverticulosis [28]. Reddy et al., from the USA, showed that NAFLD was significantly as-sociated with diverticular disorders, inflammatory bowel diseases, gallstone-related dis-eases and benign pancreatitis [22] (Figure 2).

### 2.2. Diabetes Mellitus and Diverticular Disease

The association between other comorbidities such as diabetes mellitus, hyperlipi-daemia and metabolic syndrome remain ambiguous. Different studies suggest diabetes mellitus decreases, increases or has no impact on risk of developing diverticular disease. Among the studies suggesting increased prevalence of diabetes mellitus is a French study using 70 patients with colonic diverticulum and 50 control subjects. This study reported increased prevalence of impaired glucose tolerance and hyperlipidaemia in patients with asymptomatic colonic diverticulum [29]. In concordance, a Japanese study investigating the association between colonic diverticulum and metabolic disorders used middle-aged (51–59 years) observed an elevated prevalence of type 2 diabetes mellitus and hyperten-sion in asymptomatic colonic diverticulum patients. The mechanism by which this asso-ciation exists can only be explained by the correlation of risk factors between diabetes mellitus and diverticular disease; interestingly, the same study observed the prevalence of the risk factors to be similar between colonic diverticula patients and non-diverticulosis patients [30].

In contrast, a Danish nationwide cohort study investigating the link between type 2 diabetes mellitus and diverticular disease concluded that, prior to adjustment for BMI, in-dividuals living with type 2 diabetes mellitus had higher prevalence of diverticular dis-ease. However, after adjusting for BMI, individuals with type 2 diabetes mellitus had a slightly lower risk of diverticular disease. They suggested two possible causes for the low-er risk observed in individuals with diabetes mellitus, the first being increased rate of life-style modification post type 2 diagnosis and the second being the use of metformin [31]. Metformin, which is the first line of treatment for diabetes mellitus in most Eastern coun-tries, has been found to lower the risk of acute diverticulitis compared to other oral hypo-glycaemic drugs including insulin [32]. These studies are in accordance with findings from Nikberg et al. and Kopylov et al., which suggest a negative association between dia-betes mellitus and diverticular disease after adjusting for BMI and smoking [24,33]. Nick-berg et al. also included adjustments for socioeconomic status, and concluded there is no association between socioeconomic status, type 2 diabetes mellitus and asymptomatic colonic diverticulosis [29]. Exploring this association is particularly important, as there is robust evidence to suggest that individuals belonging to lower socioeconomic group are more likely to develop type 2 diabetes mellitus compared to those with a higher socioeco-nomic profile, with recent data from the UK indicating increased mortality for those living in council homes and the unemployed [34,35].

### 2.3. Pathogenesis

The association between diverticulosis and diabetes mellitus is an area of ongoing research [28,36,37]. Congruities between the two diseases have been identified, including enteric nervous system damage due to a hyperglycaemic state, oestrogen deficiency in the female gender, living a western lifestyle, dysbiosis and visceral obesity [37,38]. A leading cause of the development of type 2 diabetes mellitus is consuming foods with a high-calorific content, which encourages the development of visceral obesity. A complication of diabetes mellitus includes intestinal peristalsis and enteric nervous system impairment, which ultimately can lead to intestinal diverticula formation [37,38]. High-calorie foods that are low in fibre content form a major part of the diets of most individuals with type 2 diabetes mellitus, this in turn results in gut dysbiosis. A detrimental consequence of dysbiosis is a breach in the protective intestinal barrier, promoting the development of diverticulosis. Furthermore, studies showed that hypoestrogenemia is a potential precursor to both diabetes mellitus and diverticulosis, as it could result in both thickening of the collagen in the muscular layer of the intestine and in visceral obesity [28,36,37].

A Mendelian randomization study was undertaken to assess the casualty of type 2 diabetes mellitus, adiposity, alcohol and coffee consumption, and smoking with relation to diverticular disease. Results of this study suggested that genetic liability to type 2 diabetes mellitus and a higher genetically predicted body mass index (BMI) in addition to initiation of smoking was found to be correlated with a higher risk of developing diverticular disease [1,38,39,40]. The calculated combined odds ratio of diverticular disease in the study was 1.23, with the 95% confidence interval being 1.14–1.33 and the *p* value < 0.001 for 1-standard deviation increase in BMI (approximately 4.8 kg/m^2^); and the calculated combined odds ratio was 1.04 with a 95% confidence interval 1.01–1.07 and a *p* value = 0.007 for 1-unit of increase in the log-transformed odds ratio of type 2 diabetes mellitus [1,38]. These results reinforce the causal association between type 2 diabetes mellitus and a higher BMI. Subsequently, this stresses the importance of controlling diabetes mellitus and maintaining a healthy BMI as prevention strategies and in the management of existing diverticular disease. Studies also showed that diabetes mellitus was a risk factor for acute diverticulitis [1,37,38,39]. 

Diverticular disease has been associated with chronic inflammation [37,38,39]. The most cogent evidence of this association, despite being indirect, is that several risk factors for diverticulitis are linked to systemic and chronic inflammation. Obesity, lack of physical activity, and consuming a western diet are thought to be caused by chronic inflammation and risk factors for diabetes mellitus and cardiovascular disease. The mentioned risk factors increase levels of biomarkers inflammation including the expression of histamine and matrix metalloproteinases [38,39]. This is associated with intestinal inflammation of diverticulitis. Inflammation of the intestinal mucosa in individuals with segmental colitis is linked to diverticular disease, and clinically and morphologically overlaps with inflammatory bowel disease. Results from a case-series suggested that individuals with diverticulosis, especially symptomatic uncomplicated diverticular disease, have a degree of microscopic intestinal inflammation [38,40] (Figure 2).

Moreover, the correlation between colonic diverticular disease and diabetes mellitus remains debatable [31]. A systematic review and meta-analysis were conducted and it examined the risk of developing diverticular disease in patients with diabetes mellitus [41]. The analysis showed that across six different studies, the risk of developing colonic diverticular disease after diagnosis of diabetes mellitus had an odds ratio of 1.25 with a confidence interval of 0.87 to 1.79 [31,41,42]. However, the findings from the individual studies of the meta-analysis were varied. Due to this, studies that were examined in the meta-analysis have shown that diabetes mellitus has either an increased, reduced, or no effect on developing diverticular disease [30,43]. Additionally, the majority of studies conducted on the matter did not identify whether type 1 or type 2 diabetes mellitus had an impact on the development of diverticular disease [43] (Table 2). Potential mechanisms that could account for a correlation between the two diseases remain unclear. Minimal dietary fibre intake, obesity and genetic factors have all been suggested as contributing factors to the development of diverticular disease [38,43]. On the contrary, conservative management including lifestyle changes and pharmacological anti-diabetic drugs have been proposed to lead to a reduced risk of developing diverticular disease [32,43,44].

### 2.4. Complications

Both diabetes mellitus and diverticular disease are individually associated with countless complications; some of these complications may exacerbate or coincide with one another in patients with both conditions [44,45]. While managing patients who suffer from both diseases, it is critical to understand and be wary of these complications [2,45].

Individuals living with diabetes mellitus tend to have a degree of chronic inflammation which can exacerbate diverticulitis. High levels of inflammatory markers including cytokines could intensify the inflammatory response in diverticular disease. Another complication includes impaired healing in diabetes mellitus [46]. As individuals with diabetes mellitus have compromised immune functions and poor blood flow, in addition to impaired wound healing, this complicates the treatment of diverticular disease, especially after surgical procedures (fistulas and perforations). As individuals with diabetes mellitus have a compromised immune system, they are at a higher risk for infections compared to the general public; these include infections associated with diverticular disease such as intra-abdominal abscesses [2,45,46]. Poor blood flow in the gastrointestinal system can also worsen diverticular disease complications such as intestinal ischemia, and this can result in devastating outcomes.

Moreover, patients with diabetes mellitus tend to be at a greater risk of developing poorer surgical outcomes [47]. Operative management of diverticular disease complications in diabetic patients was shown to be correlated with an increased incidence of acute kidney injury and infections postoperatively. Several studies revealed diabetes mellitus was a major risk factor for the development of pneumonia postoperatively following surgery for both colectomies and colonic diverticulitis [46,47,48].

These findings indicate that particular focus should be put on monitoring the patient’s blood tests, including renal function and infection markers [37,38]. Prompt management, where appropriate, should be initiated to minimize the risk of adverse effects. Despite this, diabetes mellitus was not shown to increase the risk of surgical mortality in patients undergoing surgical treatment. The study also showed that diabetic patients with diverticulitis tend to present clinically at a more advanced stage when measured using the Ambrosetti and Hinchey scoring systems. Diabetic patients hospitalized with diverticulitis were also shown to have a longer hospital stay [45,46,48].

### 2.5. Treatment

Treating patients diagnosed with both diabetes mellitus and diverticular disease can be challenging [1]. However, proposed management plans in the literature include maintaining a tight glycaemic control and making some dietary modifications. Strict glycaemic control helps in reducing the body’s inflammatory state and this in turn decreases the risk of exacerbations of diverticular disease and diverticulitis. Following up diabetic patients is essential in order to monitor their HbA1c and adjust their anti-diabetic medications accordingly, as this helps to limit the number of complications [1,33,43]. A mainstay of type 2 diabetes mellitus treatment is lifestyle modification. Studies have demonstrated that lifestyle modifications in diabetic patients, which contribute to lowering BMI, could result in lowering the risk of developing diverticular disease. These modifications include increasing physical activity and consuming a high-fibre diet [31,32,33] (Figure 2 and Table 3).

Interestingly, some research suggests that anti-diabetic drug metformin can play a protective role against the development of diverticulitis. In patients with type 2 diabetes mellitus, metformin helps in delaying the disease progression of diverticulosis and in relieving the symptoms of diverticulosis [31,32]. Metformin is a first-line anti-diabetic drug for type 2 diabetes mellitus. A study concluded that metformin use was correlated with a reduced risk of developing acute diverticulitis when compared with alternative anti-diabetic drugs [31,32,33].

Moreover, in patients with diverticular disease and diabetes mellitus, it is pivotal to balance a diet rich in fibre while simultaneously limiting carbohydrate intake. A high-fibre diet is highly recommended to help prevent diverticular disease complications, and monitoring carbohydrate consumption is important for glycaemic control [31,33]. Furthermore, having regular colonoscopies for diabetic patients with diverticular disease is important for purposes of surveillance and in addition to monitoring inflammatory markers [31]. Finally, patients with both diabetes mellitus and diverticular disease should avoid the use of corticosteroids and non-steroidal anti-inflammatory drugs (NSAIDs) for pain relief as they can increase the risk of bleeding [39,40]. Corticosteroids have been associated with immunosuppression and an increased risk of hospital admission in patients with diverticular disease [39].

**Figure 3:**
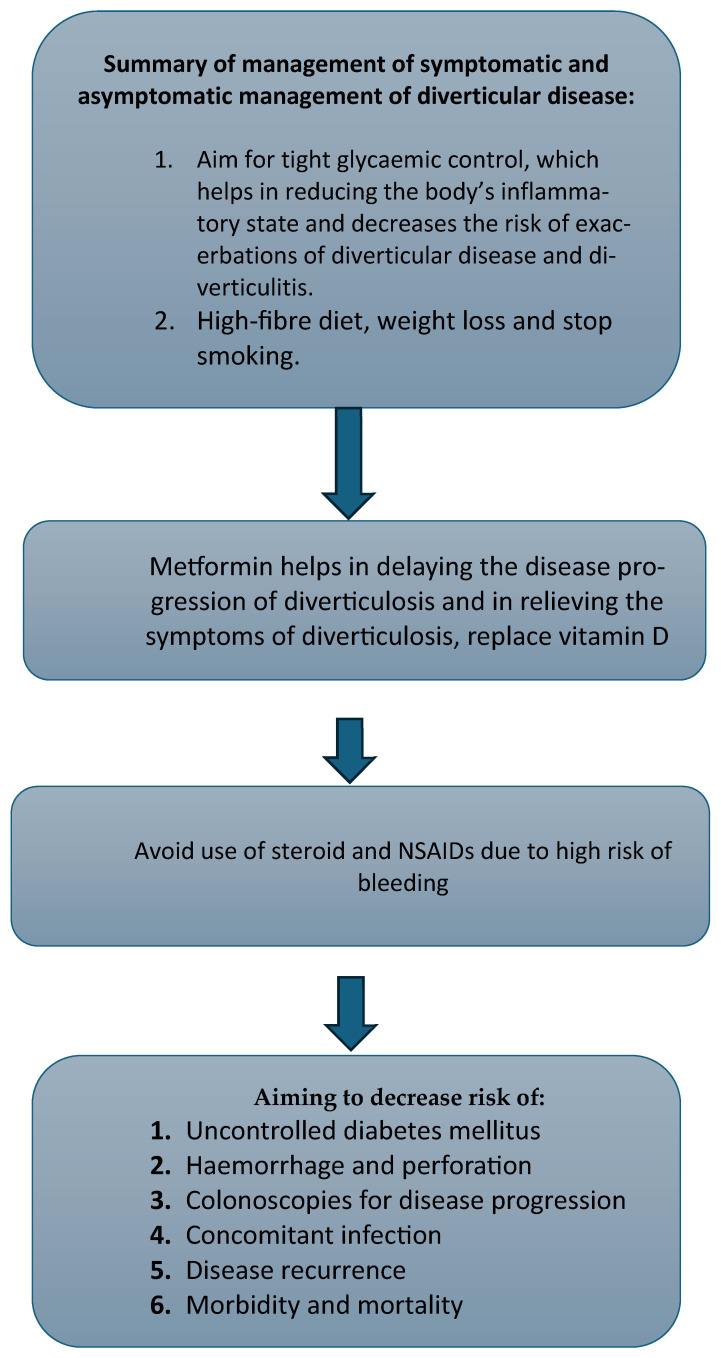
Approach for management of diverticular disease in individuals with diabetes mellitus.

For asymptomatic diverticulitis, an established management plan in the literature includes preventing recurrence or progression of the disease. This involves preventing and treating coexisting diabetes mellitus, making lifestyle changes including consuming a high-fibre diet and performing vigorous regular exercise, refraining from smoking, ensuring adequate vitamin D levels, and avoiding the use of aspirin and NSAIDs [43,49,50]. This is due to diabetes mellitus being associated with an increased risk of complicated and symptomatic diverticular disease in addition to colonic diverticular haemorrhage. Hence, it is of utmost importance to treat current diabetes mellitus and to advocate for primary prevention. Obesity and a high BMI, smoking and lower vitamin D levels can promote progression to diverticular disease of the colon and acute diverticulitis; therefore, these should be avoided [51,52,53,54] (Figure 3).

**Table 3:** The primary management strategies we recommend for patients with both diverticular disease and diabetes. This is further elaborated in the management section of this review.

Management Strategy	Conclusions	References
***Tight glycaemic control***	- Aim for tight glycaemic control; this helps in reducing the body’s inflammatory state and decreases the risk of exacerbations of diverticular disease and diverticulitis. - Metformin has been shown to decrease the risk of acute diverticulitis in patients with type 2 diabetes mellitus.	[1,33,44] [37],
***Lifestyle and dietary modifications***	- Studies have shown lowering the modifications aimed at lowering BMI could result in reducing the risk of diverticular disease. - Further studies have also shown a correlation between western diet and incidence of diverticular disease, modifying the diet to include a more prudent diet has been shown to reduce the risk of diverticular disease.	[31,32,33]
***Regular surveillance***	- Regular colonoscopies are essential for patients with both diabetes mellitus and diverticular disease. - Non-steroidal anti-inflammatories should be avoided in patients diagnosed with both diabetes mellitus and diverticular disease, as it has been shown to increase the risk of bleeding. - Use of corticosteroids in patients with diverticular disease is associated with increased hospitalization.	[50,51]

## 3. Future Directions

The pathogenesis of diverticular disease in the presence of diabetes mellitus remains an area for further investigation. It is not yet clear how diabetes mellitus modulates the cellular and molecular functions associated with diverticular disease. The efficacy of metformin as a therapeutic agent in diverticulitis warrants further prospective, randomized, and interventional investigations. The safety of other diabetes mellitus medications should also be studied in patients with both diabetes mellitus and diverticulitis. Additional studies are needed to explore the relationship between the duration of diabetes mellitus, diverticular disease, and associated risk factors. More focus should be placed on assessing the impact of newer diabetes mellitus management technologies—such as continuous glucose monitors and newer diabetes mellitus medications—for gastrointestinal health and diverticular disease risk, symptoms and outcomes. More research is needed to better understand how the progression of diabetes mellitus affects the development and severity of diverticular disease. Establishing patient education programmes that emphasize self-management strategies for both diabetes mellitus and diverticular disease is crucial.

Additionally, the development and validation of tools to predict the risk of diverticular disease in diabetic patients could help clinicians intervene early and personalize treatment. The development of interdisciplinary care approaches can address the needs of patients with both diabetes mellitus and diverticulitis. Telemedicine services could facilitate continuous monitoring and education for patients with both conditions. Moreover, more studies should be conducted to clarify the relationship between obesity and other diabetes mellitus-related risk factors and diverticular disease. Specifying dietary interventions based on individual metabolic responses can optimize management for individuals with diabetes mellitus and diverticular disease.

The potential use of oestrogen therapy as a treatment for both diabetes mellitus and diverticulitis should also be investigated.

## 4. Limitations and Strengths of This Review

One of the limitations of this narrative review is the inclusion of manuscripts published only in English, which prevents us from commenting on publications in other languages. However, the strengths of this review lie in the breadth of studies included, providing a comprehensive and up-to-date overview of the intersection between diabetes mellitus and diverticular disease. By drawing from multiple sources, this review offers a robust synthesis of existing knowledge and highlights emerging trends in this field. It includes a summary of the epidemiology, pathogenesis, and treatment of these conditions

One of the key contributions of this review is its projection of future directions and highlighting the positive association of diabetes mellitus and diverticular disease, emphasizing the need for cellular and molecular research, in addition to population-based studies. This forward-looking perspective, along with the identification of gaps in the literature, offers valuable insights for future research on the aging population living with both diabetes mellitus and diverticular disease. We also believe that the narrative style of this review will make it accessible to a wide range of healthcare professionals, including geriatricians, surgeons, diabetologists, and nutritionists. This may ultimately encourage further scientific discussion and promote interdisciplinary approaches to care for the aging population.

## 5. Conclusions

The risk factors of diabetes mellitus might be positively linked to diverticular disease. The intersection of diabetes mellitus and diverticular disease represents a growing challenge in geriatric care. Both conditions share common pathophysiological mechanisms, such as inflammation, impaired immunity, and gut dysbiosis, which are amplified in the aging population. Multidisciplinary management—including individualized nutrition plans, careful medication management, and early detection—is essential to reduce morbidity and improve outcomes. Further research into the molecular mechanisms connecting these conditions may provide new therapeutic avenues, improving the quality of life for elderly patients with diabetes mellitus and diverticular disease. Further studies are needed to explore the complex relationship between diabetes mellitus and diverticular disease, and the impact of glycaemic control on diverticular disease outcomes. Importantly, future research needs to investigate lifestyle interventions and the role of microbiome-modifying therapies. The findings of this review highlight the important role of early screening and prevention for diverticular disease in individuals with type 2 diabetes mellitus.

## Figures and Tables

**Table 1:** A more comprehensive explanation and representation of the most prevalent risk factors linked to diverticular disease.

*Risk Factor*	*Findings*	Statistical Significance	*Study Size*	*Reference*
**Obesity**	- Increased prevalence of asymptomatic diverticular disease observed with increase in body mass index. - An association between higher BMI and severity of diverticular disease exists. - Two separate studies concluded increased prevalence of diverticular disease with higher waist circumference.	- BMI 25–30 OR 3.02 (95% CI 1.33–6.88) and BMI > 30 OR 4.43 (95% CI 1.88–10.49). - OR1.44 (95% CI 1.06–1.96). - OR2.8 (95% CI 1.7–4.9) and OR2.129 (95% CI 1.005–4.510), respectively.	- 223 patients analysed (87 included). - 393 subjects, 194 incidents of diverticular disease. - Study 1: 126 with 53 diagnosed with diverticular disease. Study 2: 203 patients, 61 patients diagnosed with diverticular disease.	[13] [14] [15,16]
**Diet**	- Western diet (rich in fat, red meat and refined grains) is associated with increased incidence of diverticular disease - Total red meat consumption increased risk of incidental diverticular disease, particularly if it is unprocessed.	- HR 1.55 (95% CI, 1.20–1.99). - RR of 1.58 (95% CI: 1.19, 2.11.	- Prospective cohort study involving 46,295; 1063 incidents of diverticular disease. - Prospective study involving 46,461; 764 incidents of diverticular disease	[17] [18]
**Age**	- Increased likelihood of diverticular disease regardless of ethnicity is observed with increased age. Older patients are more likely to have left side of colon affected rather than right. Severity of diverticular disease increases with age.	- OR 2.24 (95% CI: 1.60–3.15).	- Prospective study involving 848; 103 diagnosed with diverticular disease.	[19]
**Hypertension**	- Hypertension is significantly associated with development of diverticular disease - Unregulated hypertension and the lack of anti-hypertensive management is also associated with developing diverticular disease.	- OR 1.092 (95% CI 1.007–1.185). - OR 2.07 (95% CI 1.17–3.67) and OR 1.73 (95% CI 1.21–2.75).	- Retrospective study involving 11,086 patients. - Prospective study involving 2748 patients.	[20] [21]
**Fatty liver disease**	- Non-alcoholic fatty liver disease was significantly associated with development of diverticular disease.	- OR 4.26 (95% CI 3.89–4.67).	- Retrospective study analysing 7,800,441 discharge records.	[22]
**Hyperlipidaemia**	- High LDL levels associated with increased severity of diverticular disease. - Hypertriglyceridemia is also associated with development of diverticular disease.	- OR 2.35 (95% CI 1.09–5.06). - OR 1.287 (95% CI 1.032–1.607).	- Cohort study involving 393 patients. - Cross-sectional study involving 6180 patients.	[14] [23]
**Hypothyroidism**	- Higher incidence of diverticular disease in patients with hypothyroidism.	- OR 2.403 (95% CI 1.303–4.431).	- Retrospective case control involving 3175 patients.	[24]

**Table 2:** Table 2 summarizes the key findings from the reviewed literature that demonstrate the association between diabetes and diverticular disease. It provides an overview of the evidence supporting this relationship while also outlining the limitations and challenges faced by these studies.

Study	Year	Finding	Exposure	Study Size	Limitations
***Prevalence rates of type 2 diabetes and hypertension are elevated among middle aged Japanese men with colonic diverticulum***	2007 [30]	- Type 2 diabetes mellitus more prevalent in patients with asymptomatic diverticular disease compared to the control. - The colonic diverticular are present on the right side of the colon in the Japanese population in comparison with left side for western population.	Type 2 diabetes mellitus (*p* value = 0.047) elevated in Japanese middle-aged patients with diverticular disease.	Cross-sectional study involving 954 patients.	Confounding factors such as diet, genetic predisposition that present as risk factors were not analysed completely.
***Type 2 diabetes mellitus and risk of diverticular disease: a Danish cohort study.***	2022 [31]	- Patients with type 2 diabetes mellitus had increased incidence of diverticular disease. - Post adjustment for BMI, the incidence of diverticular disease in type 2 diabetes mellitus patients decreased.	HR 0.88 (95% CI 0.80–0.96) HR 0.76 (95% CI 0.67–0.87)	Population-based study involving 15,047 patients with type 1 diabetes mellitus and 210,606 without.	Type 2 diabetes mellitus patients identified through a national registry, could include patients with late onset type 1 diabetes.
***Metformin use in diabetics with diverticular diseases is associated with reduced incidence of diverticular disease.***	2017 [32]	- Use of metformin, an oral hypoglycaemic drug, is associated with lower incidence of acute diverticulitis compared to asymptomatic diverticular disease.	40% compared to 60% respectively.	Retrospective case control study involving 174 patients.	No other confounding variable accounted for besides other oral hypoglycaemic drugs and insulin.
***Diabetes increases morbidities of colonic diverticular disease and colonic diverticular haemorrhage: A systematic review and meta analysis.***	2017 [42]	- Meta-analysis of prospective studies concluded patients with diabetes mellitus had 1.25 times higher incidence of colonic diverticular disease morbidity. - The incidence of diabetes mellitus on colonic diverticular disease is borderline in the retrospective studies.	OR 1.201 (95% CI 1.135–1.270) OR 1.176 (95% CI 0.563–2.454)	Two prospective studies included in the meta-analysis. 4 retrospective studies included in this meta-analysis.	Some of the prospective studies had randomized cohorts. Retrospective studies showed heterogeneity between diabetes and colonic diverticular disease.
***Gastrointestinal consequences of Type 2 diabetes mellitus and impaired glycaemic homeostasis: A Mendelian randomization study.***	2023 [38]	- Genetic liability to type 2 diabetes mellitus is associated with increased risk of diverticular disease.	OR 1.04 (95% CI 1.01–1.07)	Mendelian randomization study using UK Biobank, Finngen and GWAS studies.	Influence of diabetes on development of gastrointestinal conditions through other causal pathways, not possible to rule out.

## Data Availability

This review article and all information available included in this review.
